# Automated differentiation of non‐fluent and logopenic primary progressive aphasia in Italian speakers using acoustic and linguistic speech measures

**DOI:** 10.1002/dad2.70450

**Published:** 2026-08-14

**Authors:** Francesco Pierotti, Carmen Morinelli, Valentina Moschini, Sonia Padiglioni, Giulia Giacomucci, Salvatore Mazzeo, Silvestro Micera, Andrea Bandini, Valentina Bessi

**Affiliations:** ^1^ The Biorobotics Institute & the Department of Excellence for Robotics and AI Scuola Superiore Sant'Anna Pisa Italy; ^2^ SOD Neurologia Dipartimento di Neuroscienze degli Organi di Senso AOU Careggi Florence Italy; ^3^ Regional Referral Centre for Relational Criticalities Tuscany Region Florence Italy; ^4^ Department of Neuroscience, Psychology, Drug Research and Child Health University of Florence Florence Italy; ^5^ Vita‐Salute San Raffaele University Milan Italy; ^6^ IRCCS Policlinico San Donato San Donato Milanese Italy; ^7^ Modular Implantable Neuroprostheses (MINE) Laboratory Università Vita‐Salute San Raffaele Milan Italy; ^8^ Translational Neural Engineering Laboratory Neuro‐X Institute École Polytechnique Federale de Lausanne (EPFL) Lausanne Switzerland; ^9^ Department of Engineering “Enzo Ferrari” (DIEF) University of Modena and Reggio Emilia Modena Italy; ^10^ Health Science Interdisciplinary Research Center Scuola Superiore Sant'Anna Pisa Italy; ^11^ KITE Research Institute University Health Network Toronto Ontario Canada

**Keywords:** acoustic‐linguistic biomarkers, connected speech analysis, data‐driven classification, feature explainability, primary progressive aphasia

## Abstract

**Introduction:**

Differentiating the nonfluent/agrammatic and logopenic variants of primary progressive aphasia (PPA; nfvPPA and lvPPA, respectively) remains clinically challenging due to overlapping subtle speech and language impairments. We investigated whether automated connected speech analysis can support differential diagnosis.

**Methods:**

We analyzed connected speech from 84 Italian speakers with PPA (23 nfvPPA, 23 semantic variant [svPPA], and 38 lvPPA) using a picture‐description task. Prosodic, phonological, and morphosyntactic features were extracted. Machine‐learning classifiers were trained for binary (lvPPA vs. nfvPPA) and multiclass (lvPPA, nfvPPA, svPPA) classification. Explainability analyses identified key features.

**Results:**

The combination of speech and language features effectively discriminated among PPA variants. Binary classification reached 81.67% accuracy, while multiclass classification reached 63.86% accuracy. Noun rate, local jitter, articulation rate, and total pauses were among the most informative features.

**Discussion:**

Automated analysis of connected speech provides objective linguistic biomarkers that enhance diagnostic accuracy and support reliable differentiation of PPA variants in clinical practice.

## Highlights

- Automated combination of acoustic and linguistic accurately classifies primary progressive aphasia (PPA) variants.
- Noun rate and jitter emerge as key discriminative features.
- This was the first automated speech study in native Italian PPA speakers.

## 1 BACKGROUND

Primary progressive aphasia (PPA) encompasses a group of neurodegenerative syndromes characterized by an insidious emergence of language impairments and gradual worsening over time. These deficits are typically associated with degeneration of language networks involving frontal, temporal, and parietal regions of the left hemisphere.^1^ Early in the disease, language impairment appears relatively isolated, leading to limitations in communication, social participation, and functional abilities. As the disease progresses, deterioration of language function is accompanied by involvement of other cognitive domains and functional abilities.

Current classification distinguishes three main variants: semantic (svPPA), nonfluent/agrammatic (nfvPPA), and logopenic (lvPPA), each with distinct clinical, neuropsychological, imaging, and neuropathological profiles: svPPA and nfvPPA are mostly linked to frontotemporal lobar degeneration (FTLD) and lvPPA to Alzheimer's disease (AD).^2^ Clinically, lvPPA shows slow, interrupted speech with word‐finding pauses, phonological errors, and impaired sentence repetition; nfvPPA features effortful, agrammatic, and distorted speech with comprehension deficits, with syntactically complex sentence comprehension deficits and relatively preserved single‐word comprehension. svPPA presents with naming and single‐word comprehension difficulties. Despite these distinctions, overlap complicates differentiation,^3^ particularly between nfvPPA and lvPPA, which share subtle deficits—word‐finding problems, pauses, reduced fluency, and phonological or articulatory errors.^2^ Some patients meet criteria for multiple variants (“mixed PPA” [mPPA]),^4^ others are “unclassifiable PPA” (uPPA).^2^, ^5^ Nevertheless, precise identification is essential for planning targeted interventions, given the distinct progression patterns and specific needs associated with each variant.^6^

Current clinical diagnostic assessments rely on comprehensive tests evaluating both linguistic and cognitive aspects. However, these tests are typically manually analyzed, and therefore time consuming and expensive.

Recent advances in computational linguistics and artificial intelligence (AI) have opened new diagnostic perspectives. In particular, digital linguistic biomarkers (DLBs)—objective behavioral indicators obtained through digital tools—may enhance the diagnosis and classification of PPA^7^ by characterizing linguistic profiles, supporting differential diagnosis, and identifying potential biomarkers not yet included in clinical criteria.

Several studies have explored computational approaches to PPA variant classification, focusing on lvPPA and nfvPPA. Lukic et al.^8^ used a classical machine learning (ML) approach with morphosyntactic measures from picture description tasks, achieving an area under the curve (AUC) of 0.95. Rezaii et al.^9^ applied unsupervised AI based on large language models (LLMs) to classify nfvPPA, lvPPA, and svPPA with an accuracy of 97.9%. Themistocleous et al.^10^ combined acoustic and linguistic measures, applying ML algorithms to distinguish all three subtypes, achieving 80% multiclass accuracy. Other studies focused on lexical or acoustic domains or specific variants.^11^, ^12^, ^13^

However, no studies have investigated automated speech analysis in native Italian speakers with PPA. Existing literature is heterogeneous in sample size, clinical characteristics, analytical methods, and elicitation paradigms, limiting comparability and generalizability. Reliable, objective tools for differential diagnosis and subtype classification across larger populations are still lacking.

## 2 METHODS

### 2.1 PPA participants

We included individuals who attended the Cognitive Disorders and Dementia Centre (CDCD) at the Neurology Unit 1 of the Careggi University Hospital in Florence, between September 2017 and January 2025. These individuals sought care at our center due to language disorders.

All participants underwent a preliminary neurological examination aimed at collecting a complete clinical and family history and performing a general and neurological objective examination. Disease duration was defined as the time elapsed from symptom onset to baseline neuropsychological assessment. Participants were subsequently excluded following specific exclusion criteria (see Supplementary Methods §2.1 in supporting information).

Based on the results of neuropsychological and language assessments, a team of neurologists and neuropsychologists specialized in neurodegenerative disorders classified the included participants into variants according to current diagnostic and classification criteria for PPA^2^ (lvPPA, svPPA, nfvPPA or uPPA, or mPPA if not fulfilling any variant‐specific criteria).

**RESEARCH IN CONTEXT**

**Systematic review**: The authors reviewed the existing literature using traditional databases (e.g., PubMed) and recent computational studies on automated speech analysis in primary progressive aphasia (PPA_. Although machine learning applications for PPA variants discrimination are increasing, evidence remains heterogeneous in sample size, linguistic tasks, and extracted features. No prior studies have investigated native Italian speakers. Relevant and recent contributions on acoustic, linguistic, and multimodal classification approaches are appropriately discussed.

**Interpretation**: Our findings support an automated, multimodal framework including acoustic and morphosyntactic speech features to differentiate among PPA variants. The results are consistent with established clinical descriptions of PPA variants.

**Future directions**: The article proposes future validation in larger, balanced, and longitudinal cohorts, with imaging and biological markers, while exploring prognostic applications to monitor disease progression and support personalized intervention strategies.

For this study, 113 consecutive participants (mean age 69.23 ± 7.08 years; 57 females) who met the criteria for a diagnosis of PPA according to Mesulam^1^ were included. Among them, 23 were classified as nfvPPA, 38 as lvPPA, and 23 as svPPA; the remaining 29 individuals with uPPA or mPPA were excluded.

The study procedures and data analysis were performed in accordance with the Declaration of Helsinki and the ethical standards of the committee on human experimentation of our institute. The study was approved by the local institutional review board (reference 15691oss). All participants provided informed consent to participate in the study and to obtain details and results of their research published.

### 2.2 Neuropsychological and language assessment

All patients underwent a comprehensive neuropsychological assessment, including the Mini‐Mental State Examination (MMSE)^14^ as a measure of global cognitive functioning, and the Frontal Assessment Battery^15^ for the overall evaluation of executive function. Immediate and working memory for verbal and visuospatial material, attention, executive function, linguistic/executive function, learning abilities and long‐term verbal memory for verbal material, visuoconstructive abilities, long‐term visuospatial memory, and language were evaluated using various gold standard tests. A complete list of the tests used are reported in Table S1 in supporting information.

Speech samples of the figure description task of the Screening for Aphasia in NeuroDegeneration (SAND) battery^16^—connected speech—were recorded with a mobile device microphone. Performance on neuropsychological and language measures was interpreted according to the available Italian normative data and published cut‐off values.^16^

### 2.3 Audio processing and feature extraction

For each audio recording, the clinician's voice was manually removed. Acoustic and linguistic features were extracted in Python, yielding 30 prosodic, 6 phonological, and 29 linguistic measures (see Table S2 in supporting information).

**FIGURE 1:**
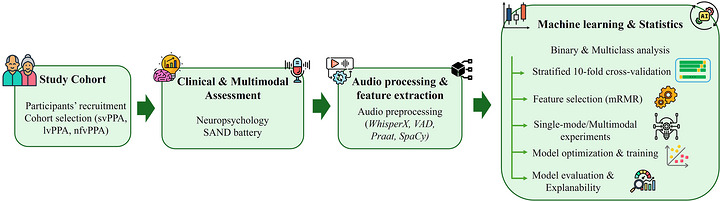
The data pipeline adopted in the study. Speech samples and clinical evaluations were collected from the recruited participants. An automated acoustic and linguistic speech processing was applied to extract several features that were used for machine learning algorithms to distinguish PPA subtypes. Explainability analysis was applied to identify the most useful features for the model prediction. lvPPA, logopenic variant primary progressive aphasia; mRMR, minimum redundancy maximum relevance; nfvPPA, non‐fluent/agrammatic variant primary progressive aphasia; SAND, Screening for Aphasia in NeuroDegeneration; svPPA, semantic variant primary progressive aphasia; VAD, voice activity detection.

The data analysis pipeline is schematized in Figure 1. Audio recordings were transcribed using WhisperX automatic speech recognition (ASR; https://github.com/m‐bain/whisper). ASR transcriptions were subsequently validated using manual transcripts (see Supplementary Methods §2.2 in supporting information).

Several speech features were extracted, including speech timings, inter‐word interval (IWI) metrics, pause metrics (in seconds), and articulation rate (words/minute). Pauses were defined as intervals > 0.05 seconds between consecutive words and categorized as short and long, using a 0.5‐second threshold.^17^ These features capture speech production, including velocity and articulatory difficulty, or inefficient word retrieval, associated with PPA.^18^, ^19^

To consider speech and pause metrics not affected by potential ASR errors, we detected the boundaries of speech in the audio signal with a voice activity detection (VAD) algorithm (https://huggingface.co/pyannote/voice‐activity‐detection), and then we computed articulation duration (in seconds), articulation rate, and pause metrics.

To extract metrics of fundamental frequency (F0), jitter, shimmer, and harmonics‐to‐noise ratio (HNR) from the entire audio recording, we exploited Praat^20^ via the Parselmouth^21^ Python interface, with sex‐specific parameter settings.

Linguistic features were extracted using Spacy (https://spacy.io/), including part‐of‐speech measures (e.g., noun and verb count and rate). A multilingual punctuation restoration model was applied to segment transcripts into sentences, enabling the extraction of sentence count, and the mean and standard deviation of words per sentence.

### 2.4 Statistical analysis

To identify differences between PPA conditions, we applied statistical analyses to demographic and neuropsychological data, as well as to acoustic and linguistic features. First, we applied the Shapiro–Wilk test to assess the normality of the data distribution, and then, depending on whether the data were normally distributed or not, we performed analysis of variance or the Kruskal–Wallis test, with post hoc Tukey honestly significant difference test or Dunn test applied if significant differences were found across the three PPA variants. Moreover, we studied the correlation between acoustic features and MMSE using age, sex, disease duration, and level of instruction as confounders. The parametric Pearson or non‐parametric Spearman correlation coefficients were computed depending on the data distribution. False discovery rate (FDR) correction was applied to account for multiple comparisons.

### 2.5 ML experiments

We trained and evaluated ML algorithms using the extracted acoustic and linguistic features. Two classifiers were trained to perform: (1) binary classification (lvPPA vs. nfvPPA), and (2) multiclass classification including svPPA. For both we used a stratified nested cross‐validation (CV) with 10 outer and 5 inner folds and a random forest (RF) classifier. At each iteration, features were *z* score normalized, and feature selection was applied on the training set to reduce the risk of overfitting and the computation cost. We first used the minimum redundancy maximum relevance (mRMR) algorithm, considering only the 10% top‐ranked features. A further stratified 3‐fold CV with an RF base estimator (n_estimators = 50) was used to identify an optimal feature subset.

For both tasks, four feature selection strategies were applied to test the ability of acoustic and linguistic features in distinguishing PPA variants:

1. Acoustic, with selection restricted to acoustic features;
2. Linguistic, with selection restricted to linguistic features;
3. Multimodal, with joint selection across all features;
4. Fusion, with independent selection within acoustic and linguistic domains, followed by a combination of selection features.

For each modality, optimal RF hyperparameters were determined via stratified 5‐fold CV, tuning the number of estimators (10, 50, 100, 150, 200) and maximum depth (none, 2, 5, 10, 20, 30). The configuration with the highest mean F1 score was selected and evaluated on the held‐out test fold.

Furthermore, for each best‐performing model in every fold, we evaluated feature importance on the test set to summarize the contribution of each feature across the entire dataset and in the prediction. Finally, we computed the cumulative matrices to assess class‐level performance and provide an aggregated overview of model behavior on the complete dataset.

## 3 RESULTS

### 3.1 Sample characteristics

Demographic characteristics are reported in Table 1. Only one significant difference was observed between the svPPA and nfvPPA for age, with the latter being slightly older (svPPA: 65.48 ± 7.73, nfvPPA: 70.91 ± 6.47). Results of ASR transcripts validation are reported in Supplementary Results §3.1 in supporting information.

**TABLE 1:** Participants’ democlinical information.

Variables	svPPA	lvPPA	nfvPPA	*p* value	*p* value svPPA vs. lvPPA	*p* value svPPA vs. nfvPPA	*p* value lvPPA vs. nfvPPA
Age (years)	65.48 ± 7.73	68.23 ± 6.90	70.91 ± 6.47	0.04	0.31	0.031	0.34
Sex				0.93	–	–	–
*Male*	11	18	12				
*Female*	12	20	11				
Education	12.57 ± 3.99	12.26 ± 5.07	10.48 ± 4.86	0.19	–	–	–
Age at onset (years)	63.45 ± 7.12	65.38 ± 7.07	67.70 ± 5.96	0.15	–	–	–
Disease duration (years)	3.24 ± 1.63	3.29 ± 2.98	3.96 ± 3.82	0.43	–	–	–

Abbreviations: lvPPA, logopenic variant primary progressive aphasia; nfvPPA, non‐fluent/agrammatic variant primary progressive aphasia; svPPA, semantic variant primary progressive aphasia.

### 3.2 Neuropsychological and language assessment

Clinical information regarding neuropsychological and language assessments is reported in Table S3 in supporting information. For the neuropsychological tests, group differences were mainly present in measures of attention, executive function, and visuo‐spatial abilities before FDR correction, and no significant differences were observed among groups in global cognitive status as measured by MMSE. After FDR correction, no features were statistically significant.

Language assessment results are summarized in Table S3. Significant group differences were observed in several naming, comprehension, repetition, reading, and connected speech measures, but none of them retained significance after FDR correction.

In further analysis, one participant in the lvPPA group was excluded due to poor audio quality.

### 3.3 Feature description statistics

**FIGURE 2:**
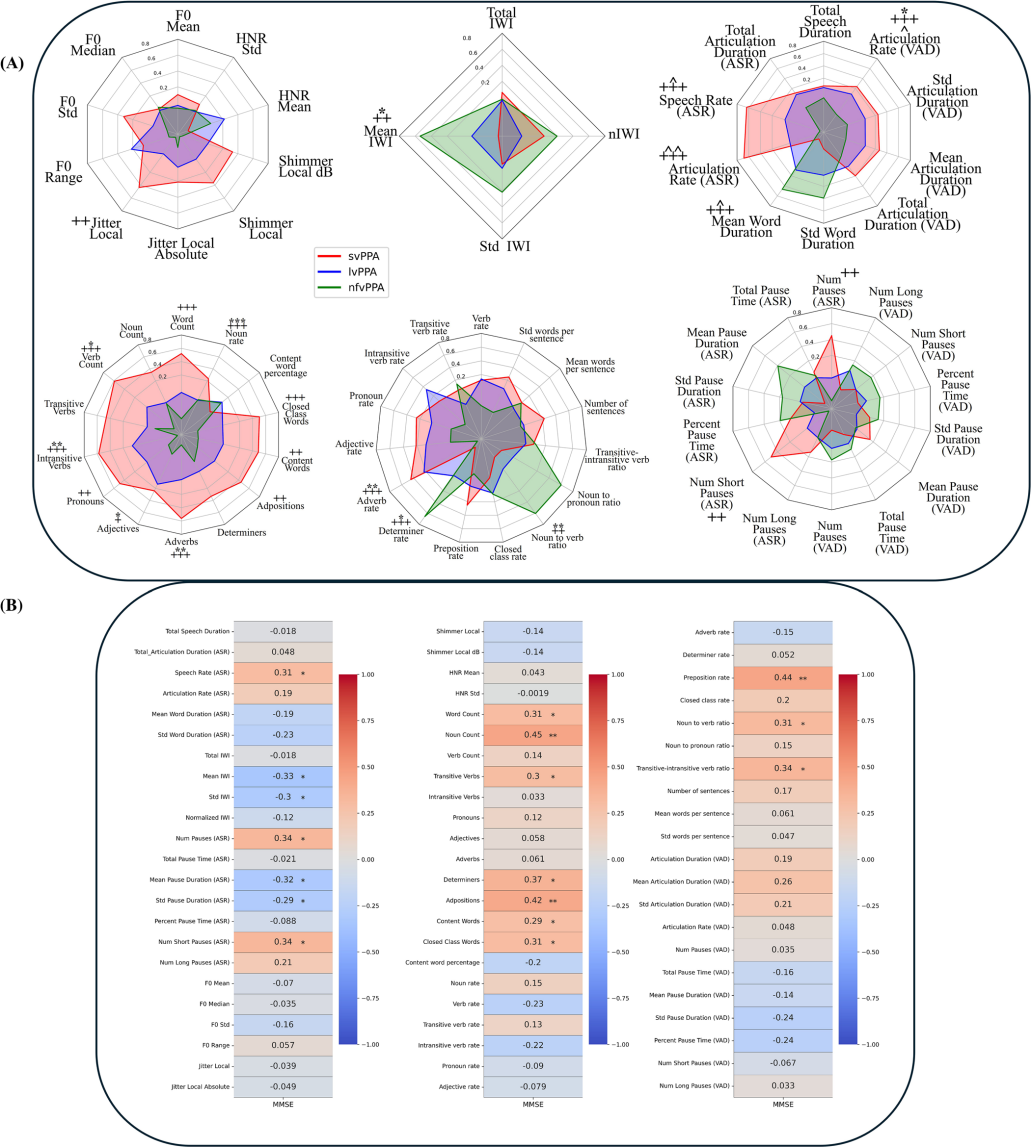
Statistical analysis results. A, Comparisons of acoustic and linguistic features between svPPA, lvPPA, and nfvPPA. Features are reported as standardized values. Statistically significant differences are expressed with * for lvPPA versus nfvPPA, + for svPPA versus nfvPPA, and ^ for svPPA versus lvPPA. B, Correlation results between acoustic and linguistic features and MMSE. Significant correlations are expressed with * for *P* < 0.05, ** for *P* < 0.01, and *** for *P* < 0.001. ASR, automatic speech recognition; F0, fundamental frequency; HNR, harmonics‐to‐noise ratio; IWI, inter‐word interval; lvPPA, logopenic variant primary progressive aphasia; MMSE, Mini‐Mental State Examination; nfvPPA, non‐fluent/agrammatic variant primary progressive aphasia; nIWI, normalized inter‐word interval; svPPA, semantic variant primary progressive aphasia; VAD, voice activity detection.

In Table S4 in supporting information and Figure 2A, we reported the results of the statistical analysis for acoustic and linguistic features. We observed several statistically significant features between svPPA and nfvPPA, while only five features were statistically significantly different between svPPA and lvPPA: mean word duration, which is higher in lvPPA, ASR speech and articulation rate, and articulation rate from the VAD algorithm, which were reduced in lvPPA. Finally, statistically significant differences were obtained between lvPPA and nfvPPA for eight linguistic features (adverbs, adjectives, adverb rate, determiner rate, intransitive verbs, noun rate, noun to verb ratio, verb count) and two acoustic features (mean IWI, articulation rate from VAD).

Correlation analysis results between MMSE and acoustic and linguistic features are reported in Figure 2B. Statistically significant positive correlations were observed with speech rate, number of total and short pauses, word count, noun count, transitive verbs, determiners, adpositions, content words, closed class words, preposition rate, noun to verb ratio, and transitive–intransitive verb ratio. On the other hand, statistically significant negative correlations were obtained with mean and standard deviation of IWI, and mean and standard deviation of pause duration from ASR.

### 3.4 Binary classification

**FIGURE 3:**
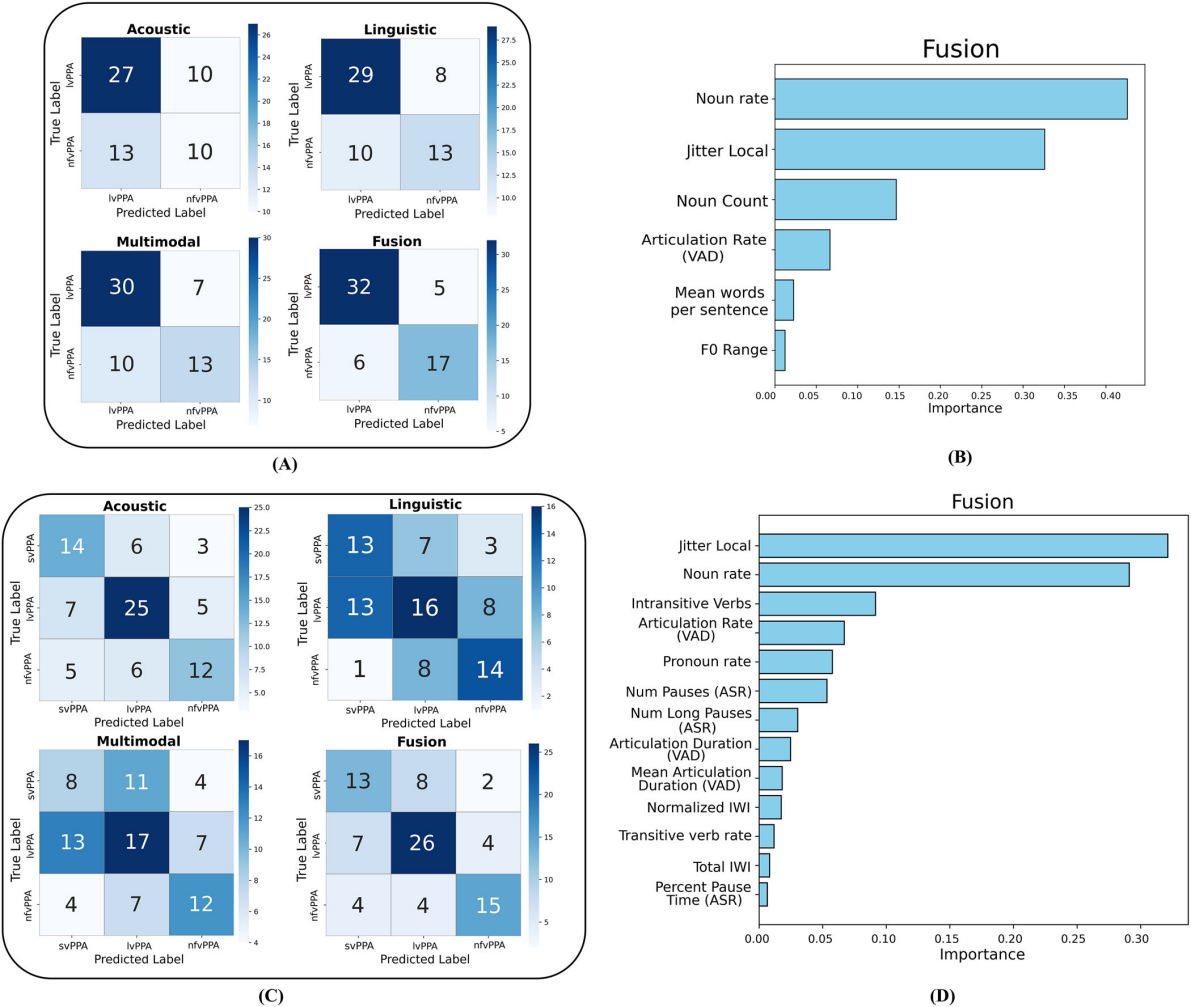
Cumulative classification results. A, Confusion matrices of all four modalities (acoustic, linguistic, multimodal, fusion) for binary classification (lvPPA vs. nfvPPA). B, The feature importance of the best resulting modality (fusion) for binary classification. C, Confusion matrices of all four modalities (acoustic, linguistic, multimodal, fusion) for multiclass classification (svPPA vs. lvPPA vs. nfvPPA). D, The feature importance of the best resulting modality (fusion) for multiclass classification. ASR, automatic speech recognition; F0, fundamental frequency; IWI, inter‐word interval; lvPPA, logopenic variant primary progressive aphasia; nfvPPA, non‐fluent/agrammatic variant primary progressive aphasia; svPPA, semantic variant primary progressive aphasia; VAD, voice activity detection.

For the binary classification (chance level 61.67%), we focused only on participants with lvPPA and nfvPPA, with speech recordings lasting 96.05 ± 37.73 and 91.14 ± 23.70 seconds, respectively. The cumulative performance (in %) and the relative confusion matrices for each modality are reported in Table 2 and Figure 3A, respectively.

**TABLE 2:** Binary classification results (lvPPA vs. nfvPPA) in %.

Modality	Accuracy	F1 score	ROC AUC	Precision	Specificity	Sensitivity
Acoustic	61.67	58.32	71.45	58.75	72.97	43.48
Linguistic	70	67.7	66.16	68.13	78.38	56.52
Multimodal	71.67	69.19	78.61	70	81.08	56.52
*Fusion*	81.67	80.44	82.14	80.74	86.49	73.91

Abbreviations: lvPPA, logopenic variant primary progressive aphasia; nfvPPA, non‐fluent/agrammatic variant primary progressive aphasia; ROC AUC, receiver operating characteristic area under the curve.

The model with both acoustic and linguistic features selected separately (i.e., fusion modality) yielded the highest performance, achieving 81.67% accuracy, 82.14% receiver operating characteristic (ROC AUC), 86.49% specificity, and 73.91% sensitivity. The explainability analysis for this modality (Figure 3B) showed that the optimal predictors were the noun rate, jitter local, noun count, articulation rate from VAD, mean number of words per sentence, and the F0 range. This analysis revealed that the highest contribution was given by the noun rate and local jitter, which were the only ones selected in each fold. Among these features, only the noun rate and the articulation rate (computed with VAD) were statistically significant between lvPPA and nfvPPA, with higher values of noun rate and lower values of articulation rate associated with the nfvPPA group.

The acoustic modality achieved the lowest classification accuracy. Specifically, it yielded an accuracy of 61.67% and a ROC‐AUC of 71.45%. The linguistic strategy achieved an accuracy of 70%, with a ROC‐AUC of 66.16%. Finally, the Multimodal approach showed an accuracy of 71.67% and a ROC AUC of 78.6%.

### 3.5 Multiclass classification

In the multiclass analysis (chance level 44.58%), svPPA was included, with a speech recording lasting 98.13 ± 41.86 seconds. The cumulative performance (in %) and the confusion matrices relative to each modality are reported in Table 3 and Figure 3C, respectively. Overall, the best classification performance across the three classes was achieved by the fusion modality, with an accuracy of 65.06% and ROC AUC of 76.73%. For the class‐specific metrics, the fusion modality yielded the highest results for all (svPPA, lvPPA, and nfvPPA). Specifically, it showed precisions of 54.17%, 68.42%, and 71.43% for svPPA, lvPPA, and nfvPPA; specificities of 81.67%, 73.91%, and 90.00%; and sensitivities of 56.52%, 70.27%, and 64.22%, respectively. The overall performance of acoustic modality achieved an accuracy of 61.45% with a ROC AUC of 70.37%. Instead, the linguistic and multimodal approaches achieved lower results, showing an overall accuracy of 51.81% for linguistic and 44.58% for multimodal, and a ROC AUC of 70.33% and 64.12%, respectively.

**TABLE 3:** Multiclass classification results (svPPA vs. lvPPA vs. nfvPPA) in %.

Modality	Overall accuracy		Overall F1 score	Overall ROC AUC	Class	Precision	Specificity	Sensitivity
Acoustic	61.45	60.45		70.37	svPPA	53.85	80	60.87
					lvPPA	67.57	73.91	67.57
					nfvPPA	60	86.67	52.17
Linguistic	51.81	52.46		70.33	svPPA	48.15	76.67	56.52
					lvPPA	51.61	67.39	43.24
					nfvPPA	56	81.67	60.87
Multimodal	44.58	44.26		64.12	svPPA	32	71.67	56.52
					lvPPA	48.57	60.87	51.35
					nfvPPA	52.17	81.67	34.78
*Fusion*	65.06	64.28		76.73	svPPA	54.17	81.67	56.52
					lvPPA	68.42	73.91	70.27
					nfvPPA	71.43	90	65.22

Abbreviations: lvPPA, logopenic variant primary progressive aphasia; nfvPPA, non‐fluent/agrammatic variant primary progressive aphasia; ROC AUC, receiver operating characteristic area under the curve; svPPA, semantic variant primary progressive aphasia.

The features that mainly contributed to the classification in the best modality (i.e., fusion; Figure 3D) were jitter local and noun rate, which were the two most important features also in the binary classification. Also, the articulation rate from VAD contributed to distinguishing the three classes, as was also present in the binary classification. Notably, temporal prosodic measures such as IWI and pause metrics contributed to the classification, with a higher number of pauses, and total and mean articulation duration associated with svPPA. Moreover, other linguistic features that contributed to the classification were intransitive verbs, pronoun rate, and transitive verb rate.

## 4 DISCUSSION

This study examined the contribution of acoustic and linguistic features derived from connected speech in effective differentiation among PPA variants, demonstrating that a multimodal approach can reliably distinguish variants and provide objective, interpretable speech biomarkers reflecting prosodic, articulatory, and morphosyntactic disruption. By combining these features in an automated pipeline with cross‐validated ML and explainability analyses, this work advances the growing body of computational approaches for PPA classification while addressing some gaps identified in previous literature, including a dataset of considerable size, ecologically valid tasks, and clinically interpretable computational measures. Our findings highlight the value of integrating both prosodic and morphosyntactic information for automated assessment.

### 4.1 lvPPA versus nfvPPA: acoustic and linguistic feature fusion improves differentiation

In the binary classification of lvPPA versus nfvPPA, the model combining acoustic and linguistic features achieved an 81.67% accuracy and 82.14% ROC AUC, with 86.49% specificity and 73.91% sensitivity. Features such as noun rate and count, local jitter, and articulation rate are particularly informative, highlighting the complementary information of temporal–prosodic and linguistic measures. In particular, local jitter reflects cycle‐to‐cycle variability in vocal fundamental frequency and may index instability in phonatory and neuromuscular control of speech production.^22^ This supports a growing consensus that multimodal approaches can capture the complex interaction of lexical, syntactic, and motor‐speech impairment in PPA,^10^ and extend prior research using computational approaches to PPA. Lukic et al.^8^ demonstrated the power of morphosyntactic features in discriminating lvPPA and nfvPPA (AUC = 0.95). Indeed, we observed a strong contribution from linguistic features, particularly noun rate and count, and the mean words per sentence. These findings align with previous reports of agrammatic simplification and reliance on content words, particularly nouns, in connected speech.^23^ Moreover, our results parallel the work of Rezaii, et al.^9^ who used LLM‐based embedding analyses to classify PPA variants with accuracy approaching 98%. While their approach relied largely on semantic and syntactic embeddings, our feature‐based method identifies specific acoustic and linguistic aspects driving classification, offering a more interpretable alternative for clinical deployment. Indeed, the reduction in articulation rate reflects the hallmark motor–speech and apraxic features of the nfvPPA variant, consistent with the clinical description provided by Gorno‐Tempini et al.^2^ In contrast, lvPPA participants produced fewer nouns, but maintained a higher proportion of verbs and function words, which may reflect subtle deficits in lexical retrieval and sentence‐level planning rather than motor–speech impairment. While obtaining similar performance on classification (80% accuracy), Themistocleous et al.^10^ exploited a multimodal approach, but extracted acoustic features only for specific pronounced vowels in the speech recording.

From statistical analysis, several other linguistic features contributed to characterizing the two variants. Verb count and intransitive verb usage were all lower in nfvPPA than in lvPPA, highlighting the restricted syntactic complexity and reduced propositional content characteristic of nfvPPA speech. The use of adverbs and determiners was also reduced in nfvPPA relative to lvPPA, reflecting a more general reduction in grammatical function elements. These patterns are consistent with previous computational analyses of naturalistic speech in PPA, which report that nfvPPA is marked by a shift toward content‐dominated speech and simplified syntactic structures.^11^, ^12^ Notably, even though they were not statistically different between lvPPA and nfvPPA, local jitter and F0 range contributed substantially to the classification.

### 4.2 svPPA versus lvPPA versus nfvPPA: prosodic and lexical features distinguish variants

When including svPPA in a multiclass classification, the fusion approach again performed best, reaching overall metrics of 65.06% accuracy, and 76.73% ROC AUC, with the strongest discrimination observed for all three PPA variants. Exploring the contribution of features in the classification, local jitter and noun rate remained the most informative predictors, confirming their relevance in capturing prosodic instability and lexical distribution differences. Moreover, articulation rate, IWI, and pause metrics contributed to distinguishing the three classes, reflecting the differential impact on speech time disruption. Particularly, svPPA showed a higher articulation rate and number of overall and short pauses (but with a shorter mean duration). Statistical analyses aligned with the classification patterns. Specifically, the observed patterns indicate that nfvPPA speech is marked by agrammatic simplification and reliance on content words, svPPA by lexical–semantic retrieval deficits, and lvPPA by subtle temporal–prosodic alterations with preserved syntactic structure.

### 4.3 Clinical interpretation: automated speech features capture variant‐specific deficits

In our sample, the three PPA groups were comparable in education and sex distribution, with the only significant difference observed between svPPA and nfvPPA for age. This slight age difference should be taken into account when interpreting cognitive and language performance, although previous studies suggest that the observed differences in PPA profiles are unlikely to be solely driven by age.^2^

Neuropsychological testing in our sample revealed that the groups did not differ in overall cognitive severity, as indicated by MMSE scores. Nevertheless, group differences emerged in specific cognitive domains: svPPA performed better in attention, working memory, and processing speed, while lvPPA showed greater impairment in immediate story recall and visuospatial memory compared to nfvPPA, consistent with its relative cognitive profile.^3^, ^5^

Language assessment confirmed typical PPA profiles: nfvPPA performed better than svPPA in naming, comprehension, non‐word reading, and repetition, while sentence repetition was more impaired in lvPPA. LvPPA also scored higher than svPPA in comprehension but lower than nfvPPA in non‐word reading, illustrating subtle overlaps across variants. Connected speech analysis showed that nfvPPA produced a higher proportion of nouns, whereas lvPPA produced fewer total words, capturing variant‐specific patterns in fluency and lexical production.

Overall, these results indicate that, although the neuropsychological profiles of PPA variants largely align with expectations, conventional connected speech assessment and scoring did not reveal clear distinctions between groups. This highlights the need for more sensitive, data‐driven approaches, motivating the use of acoustic and linguistic features in an automated ML framework to achieve reliable differentiation.

Correlation analyses indicated that the features most predictive of PPA variants were also associated with global cognitive performance, suggesting that more fluent and structured speech reflects better overall cognitive functioning. However, in PPA, MMSE scores are influenced by language impairment, limiting their reliability as a measure of global cognition; therefore, some observed correlations may reflect the severity of language deficits rather than true cognitive decline.

### 4.4 Strengths and limitations

Our results support multimodal ML models for PPA classification and identify speech and language features as potential subtype markers complementing standard neuropsychological evaluations.

Strengths of this study include the integration of acoustic and linguistic features to capture both motor–speech and language aspects during naturalistic connected speech, minimizing any confounding factors that may affect acoustic measures (e.g., reading biases, task‐induced prosodic alterations). Moreover, the use of feature selection and cross‐validation ML algorithms enables the identification of the most informative features for the classification, reducing the risk of overfitting, providing robust and generalizable performance metrics, linking speech alterations to clinical evidence.

This study presents some limitations: the sample size, which, although it is in line with some previous studies,^8^, ^11^, ^17^ remains relatively modest, and the unbalanced dataset, which might influence the overall estimation. Moreover, automatic transcription may not fully capture pathological speech patterns, tending to normalize disfluencies, partial words, and mispronunciations, affecting the accuracy of transcript‐derived features. Because the linguistic features extracted in this study are lexical and syntactic in nature, this limitation has a circumscribed impact on the present analyses; however, future studies extracting phonetic or disfluency‐based measures should consider manual validation or dedicated pathological speech ASR models.

Future studies should validate these findings in larger and balanced cohorts, and consider other aspects of speech production (e.g., formants, articulatory) and multimodal datasets (e.g., integrating imaging data or video recording), which could further clarify the involved neural and motor processes and mechanisms impaired. Additionally, future cross‐linguistic and multilingual studies are essential to validate and propose speech biomarkers, with multicenter studies essential for broader validation and enhanced robust biomarker identification. Finally, longitudinal studies will enable the possibility of monitoring and eventually predicting the progression of the impairment over time, enabling an early detection of the functional decline and thus facilitating early and personalized disease management.

## AUTHOR CONTRIBUTIONS

F.P.: data curation, methodology, formal analysis, data interpretation, initial draft preparation, review, and editing; C.M.: data collection, data curation, data interpretation, initial draft preparation, review, and editing; V.M.: data collection, data interpretation, review, and editing; S.P., G.G., S.V., S.M.: review and editing; A.B., V.B.: conceptualization, data interpretation, review, and editing.

## CONFLICT OF INTEREST STATEMENT

The authors report no conflicts of interest. Author disclosures are available in the Supporting Information.

## CONSENT STATEMENT

All participants provided informed consent to participate in the study and to obtain details and results of their research published.

## Supporting information



Supporting Information

Supporting Information
