# Supplementary material for: Automated differentiation of non‐fluent and logopenic primary progressive aphasia in Italian speakers using acoustic and linguistic speech measures
Source: Alzheimers Dement (Amst). 2026 Aug 14;18(3):e70450. doi: 10.1002/dad2.70450 (PMC13474157; doi:10.1002/dad2.70450)
Supplement: Supplementary file 2 [file DAD2-18-e70450-s002.docx]

**2. Methods**

**2.1 PPA participants**

Participants were excluded from the study based on these criteria: (1) history of head trauma, neurological and/or systemic comorbidities, symptoms of psychosis, major depression, alcoholism, or substance abuse; (2) severe and early episodic memory deficits or visuoperceptual deficits; (3) severe and early behavioural disturbances; (4) non-native Italian speakers; (5) severe cognitive impairment or major language deficits preventing the conduction of an extended neuropsychological evaluation. Three patients met criterion (5) and were excluded. These cases were categorized as unclassifiable PPA (uPPA), as the severity of language deficits precluded reliable classification into a specific PPA variant.

For the purpose of group comparisons, 29 patients classified as uPPA or mPPA were not included in the statistical analyses, which were restricted to the canonical PPA variants (nfvPPA, lvPPA, svPPA) to ensure homogeneity and interpretability of the analytical groups.

In our sample, all participants underwent comprehensive neuropsychological and language-specific assessments and brain magnetic resonance imaging (MRI) or computer tomography (CT). Functional imaging with ^18^F-fluorodeoxyglucose positron emission tomography (^18^FDG-PET) was conducted in 70 participants (31 lvPPA, 22svPPA, 17 nfvPPA). Biomarker investigations for AD, cerebrospinal fluid (CSF), and/or amyloid PET, were performed in 77 participants (35 lvPPA, 22 svPPA, 20 nfvPPA). Seventy-four patients (38 lvPPA, 22 svPPA, 23 nfvPPA) gave further informed consent for genetic analysis of Apolipoprotein E (APOE), amyloid precursor protein (APP), presenilin 1 (PSEN1), presenilin 2 (PSEN2), progranulin (GRN), microtubule-associated protein tau (MAPT), and chromosome 9 open reading frame 72 (C9orf72).

**2.2 ASR transcripts validation**

To assess transcription quality, word error rate (WER), percentage of correctly transcribed words, and substitution rate were computed between manual and ASR transcripts for all participants.

**3. Results**

**3.1 ASR transcript validation scores**

Median WER was 22.72% overall (21.69% for svPPA, 24.35% for lvPPA, and 20.16% for nfvPPA), with a mean percentage of correctly transcribed words of 80.9% and a substitution rate of 6.98%.

**Table S1 Neuropsychological, language-specific tests**

| **Test** | **Aim** |
| --- | --- |
| Mini-Mental State Examination | To measure global cognitive functioning |
| Frontal Assessment Battery | To evaluate executive functioning |
| Digit Span forward | To assess of immediate and working memory for verbal and visuospatial material |
| Digit span backward |  |
| Spatial span forward |  |
| Spatial span backward |  |
| Attentive Matrices |  |
| Trail-Making Test part A | To assess attention |
| Trail-Making Test part B | To assess executive function |
| Trail-Making Test part B - Trail-Making Test part A | To assess attention and executive function |
| Trail-Making Test part B/Trail-Making Test part A |  |
| Semantic fluency | To assess linguistic/executive function |
| Phonemic fluency |  |
| Stroop Test | To assess executive function |
| Rey Auditory Verbal Learning Test | To evaluate learning abilities and long-term verbal memory for verbal material |
| Babcock Story recall Test |  |
| Rey-Osterrieth Complex Figure | To evaluate visuoconstructive abilities and long-term visuospatial memory |
| Screening for Aphasia in NeuroDegeneration | To assess language. Metrics: naming, single-word comprehension, word reading, nonword reading, nonword repetition, word repetition, sentence repetition, sentence comprehension, semantic association, informational units; total word count; noun-to-total words ratio; verb-to-total words ratio; total number of syntactic structures; ratio of subordinate clauses to total syntactic structures; ratio of self-corrected sequences to total words; ratio of phonological errors to total words; ratio of lexico-semantic errors to total words. From written composition task: number of informational units; total word count; noun-to-total words ratio; verb-to-total words ratio; ratio of correct syntactic structures to total syntactic structures; number of spelling errors; and number of lexical errors relative to total words. |

**Table S2 Acoustic and linguistic features description**

| **Speech levels** | **Variables** | **Metrics** | **Definitions** |
| --- | --- | --- | --- |
| Prosody | F0 (Hz) | Mean, median, standard deviation, range | Fundamental frequency captures pitch level |
|  | Speech duration (seconds) | Total | Speaking time in seconds, including pauses |
|  | Speech rate (words/minute) | Rate | Number of words divided by speaking duration in minutes |
|  | Articulation duration | Total (ASR), total (VAD) | Total duration of articulated speech in seconds, excluding pauses |
|  | Articulation rate (words/minute) | Value (ASR), value (VAD) | Number of words divided by articulated speech duration in minutes |
|  | Word duration (seconds) | Mean, standard deviation | Duration of ASR-recognized words |
|  | IWI (seconds) | Total, mean, standard deviation, ratio between total and total articulation duration | Temporal spacing between two consecutive words |
|  | Pauses (seconds) | Total occurrences (ASR), total occurrences (VAD), total duration (ASR), total duration (VAD), mean duration (ASR), mean duration (VAD), standard deviation (ASR), standard deviation (VAD), percentage (ASR), percentage (VAD) | Unvoiced time interval higher than 0.05 seconds |
|  | Short pauses | Total occurrences (ASR), total occurrences (VAD) | Pauses lasting less than 0.5 seconds |
|  | Long pauses | Total occurrences (ASR), total occurrences (VAD) | Pauses lasting more than 0.5 seconds |
| Phonatory | Jitter local | Value | Average difference between consecutive periods, divided by the average period |
|  | Jitter local absolute | Value | Average absolute difference between consecutive periods, divided by the average period |
|  | Shimmer local | Value | Average absolute difference between the amplitudes of consecutive periods, divided by the average amplitude |
|  | Shimmer local dB | Value | Average absolute difference in Db between the amplitudes of consecutive periods, divided by the average amplitude |
|  | HNR | Mean, standard deviation | The amplitude of the tonal relative to the noise components |
| Informativess | Word count | Value | Total number of words |
|  | Number of sentences | Value | Total number of sentences |
|  | Words per sentence | Mean, standard deviation | Distribution of sentence length |
|  | Content words | Value, percentage | Number of content words (nouns, verbs, adjectives, adverbs) |
|  | Closed class words | Value, rate | Number of function words |
| Lexico-semantic | Nouns | Count, rate | Number of nouns |
|  | Verbs | Count, rate | Number of verbs |
|  | Transitive verbs | Count, rate | Number of verbs that take a direct object |
|  | Intransitive verbs | Count, rate | Number of verbs that do not take a direct object |
|  | Pronouns | Count, rate | Number of pronouns |
|  | Adjectives | Count, rate | Number of adjectives |
|  | Adverbs | Count, rate | Number of adverbs |
|  | Determiners | Count, rate | Number of determiners |
|  | Adpositions | Count | Number of pre-/post-positions |
|  | Prepositions | Rate | Number of prepositions |
| Morphosyntactic | Noun to verb | Ratio | Ratio of noun to verbs |
|  | Noun to pronoun | Ratio | Ratio of noun to pronoun |
|  | Transitive-intransitive verb | Ratio | Ratio of transitive to intransitive verbs |

VAD: voice activity detection; ASR: automatic speech recognition; IWI: inter-word interval; HNR: harmonic-to-noise ratio.

**Table S3 Statistical results of neuropsychological features.**

| **Features** | **svPPA** | **lvPPA** | **nfvPPA** | **p-value** | **p-value corrected** | **p-value svPPA vs. lvPPA** | **p-value svPPA vs. nfvPPA** | **p-value lvPPA vs. nfvPPA** |
| --- | --- | --- | --- | --- | --- | --- | --- | --- |
| MMSE | 22.04 ± 6.64 | 19.16 ± 6.52 | 21.61 ± 6.11 | 0.14 | 0.26 | — | — | — |
| FAB | 11.26 ± 4.45 | 8.91 ± 4.05 | 9.68 ± 5.35 | 0.23 | 0.33 | — | — | — |
| Digit span forward | 4.95 ± 1.36 | 3.86 ± 0.90 | 3.95 ± 1.15 | 0.012* | 0.067 |  |  |  |
| Digit span backward | 3.16 ± 1.72 | 2.50 ± 1.18 | 2.59 ± 1.57 | 0.18 | 0.30 | — | — | — |
| Spatial span forward | 4.15 ± 1.56 | 3.58 ± 0.98 | 4.18 ± 1.37 | 0.041* | 0.13 | — | — | — |
| Spatial span backward | 3.17 ± 1.86 | 3.28 ± 1.14 | 3.24 ± 1.39 | 0.79 | 0.85 | — | — | — |
| Attentive Matrices | 37.14 ± 14.50 | 30.31 ± 11.46 | 32.18 ± 10.78 | 0.14 | 0.26 | — | — | — |
| TMT-A (s) | 69.52 ± 33.59 | 164.09 ± 208.56 | 141.60 ± 164.99 | 0.028* | 0.11 | — | — | — |
| TMT-B (s) | 156.21 ± 76.79 | 247.56 ± 107.85 | 198.00 ± 97.74 | 0.11 | 0.23 | — | — | — |
| TMT (B–A) | 105.07 ± 66.93 | 180.22 ± 97.15 | 129.00 ± 81.77 | 0.14 | 0.26 | — | — | — |
| TMT (B/A) | 3.00 ± 1.03 | 3.75 ± 1.17 | 2.79 ± 0.85 | 0.19 | 0.30 | — | — | — |
| Stroop time (s) | 85.81 ± 206.69 | 93.87 ± 168.26 | 83.09 ± 59.62 | 0.006** | 0.058 | — | — | — |
| Stroop Errors | 61.88 ± 213.08 | 46.89 ± 177.66 | 6.36 ± 8.61 | 0.077 | 0.19 | — | — | — |
| Phonemic fluency | 17.10 ± 11.72 | 17.25 ± 10.34 | 12.24 ± 7.93 | 0.19 | 0.30 | — | — | — |
| Semantic fluency | 16.48 ± 9.82 | 19.87 ± 11.13 | 25.55 ± 11.83 | 0.044* | 0.13 | — | — | — |
| RAVLT acquisition | 25.41 ± 10.97 | 21.90 ± 9.62 | 22.29 ± 8.99 | 0.72 | 0.80 | — | — | — |
| RAVLT primacy effect | 0.31 ± 0.18 | 0.33 ± 0.27 | 0.27 ± 0.18 | 0.82 | 0.87 | — | — | — |
| RAVLT recency effect | 0.545 ± 0.165 | 0.471 ± 0.294 | 0.451 ± 0.137 | 0.63 | 0.76 | — | — | — |
| RAVLT delayed recall | 3.76 ± 3.23 | 3.86 ± 3.27 | 4.50 ± 2.69 | 0.62 | 0.76 | — | — | — |
| RAVLT recognition (accuracy) | 0.84 ± 0.15 | 0.92 ± 0.05 | 0.76 ± 0.29 | 0.26 | 0.36 | — | — | — |
| RAVLT false recognitions | 4.67 ± 6.06 | 2.80 ± 2.71 | 4.00 ± 4.42 | 0.99 | 0.99 | — | — | — |
| Babcock Story recall – immediate | 4.43 ± 3.56 | 3.57 ± 2.86 | 6.63 ± 4.21 | 0.045* | 0.13 | — | — | — |
| Babcock Story recall – delayed | 5.21 ± 5.62 | 4.43 ± 3.84 | 8.13 ± 5.62 | 0.10 | 0.23 | — | — | — |
| ROCF recall | 8.97 ± 7.52 | 4.29 ± 3.58 | 8.74 ± 5.19 | 0.017* | 0.076 | — | — | — |
| ROCF copy | 27.55 ± 9.50 | 30.60 ± 56.38 | 23.39 ± 7.19 | 0.039* | 0.13 | — | — | — |
| SAND – Naming total | 5.13 ± 5.31 | 7.80 ± 4.45 | 9.72 ± 3.79 | 0.016* | 0.076 | — | — | — |
| SAND – Naming living | 2.50 ± 2.56 | 3.82 ± 2.34 | 4.74 ± 2.04 | 0.011* | 0.066 | — | — | — |
| SAND – Naming non-living | 2.63 ± 2.80 | 3.99 ± 2.30 | 5.20 ± 1.94 | 0.009** | 0.066 | — | — | — |
| SAND – Sentence comprehension | 6.17 ± 2.68 | 5.11 ± 2.47 | 6.09 ± 1.64 | 0.059 | 0.15 | — | — | — |
| SAND – Single-word comprehension total | 8.00 ± 3.73 | 10.68 ± 1.64 | 10.74 ± 2.13 | 0.005** | 0.058 | — | — | — |
| SAND – Single-word comprehension living | 4.00 ± 1.96 | 5.34 ± 0.87 | 5.35 ± 1.20 | 0.005** | 0.058 | — | — | — |
| SAND – Single-word comprehension non-living | 4.00 ± 1.91 | 5.34 ± 1.01 | 5.39 ± 1.09 | 0.006** | 0.058 | — | — | — |
| SAND – Word repetition | 5.91 ± 0.28 | 5.66 ± 0.66 | 5.30 ± 1.20 | 0.046* | 0.13 | — | — | — |
| SAND – Non-word repetition | 2.09 ± 1.21 | 1.89 ± 1.23 | 1.17 ± 1.31 | 0.031* | 0.11 | — | — | — |
| SAND – Sentence repetition | 2.83 ± 1.55 | 1.47 ± 1.21 | 2.17 ± 1.86 | 0.006** | 0.058 | — | — | — |
| SAND – Word reading | 10.43 ± 2.18 | 10.54 ± 2.40 | 10.14 ± 2.18 | 0.52 | 0.66 | — | — | — |
| SAND – Non-word reading | 6.57 ± 14.40 | 3.30 ± 1.11 | 2.55 ± 1.30 | 0.006** | 0.058 | — | — | — |
| SAND Writing –Informative Units | 3.50 ± 2.52 | 2.97 ± 1.81 | 3.67 ± 2.03 | 0.45 | 0.58 | — | — | — |
| SAND Writing – Total words | 16.00 ± 10.26 | 17.61 ± 12.70 | 20.82 ± 14.62 | 0.71 | 0.80 | — | — | — |
| SAND Writing – Nouns ratio | 0.33 ± 0.24 | 0.23 ± 0.08 | 0.29 ± 0.15 | 0.45 | 0.58 | — | — | — |
| SAND Writing – Verbs ratio | 0.164 ± 0.093 | 0.202 ± 0.084 | 0.189 ± 0.068 | 0.39 | 0.53 | — | — | — |
| SAND Writing – Syntactic structure ratio | 0.807 ± 0.364 | 0.693 ± 0.381 | 0.703 ± 0.382 | 0.37 | 0.52 | — | — | — |
| SAND Writing – Orthographic errors | 1.16 ± 2.01 | 1.41 ± 2.28 | 2.94 ± 3.64 | 0.20 | 0.32 | — | — | — |
| SAND Writing – Lexical errors | 0.51 ± 2.00 | 0.35 ± 1.73 | 0.01 ± 0.03 | 0.16 | 0.28 | — | — | — |
| SAND – Semantic associations | 1.87 ± 1.26 | 2.31 ± 1.33 | 2.57 ± 1.35 | 0.21 | 0.31 | — | — | — |
| SAND Picture description – Informative Units | 4.74 ± 2.33 | 4.92 ± 1.85 | 5.09 ± 2.00 | 0.92 | 0.94 | — | — | — |
| SAND Picture description  – Words | 119.05 ± 58.90 | 86.85 ± 32.94 | 85.78 ± 45.61 | 0.032* | 0.11 | — | — | — |
| SAND Picture description –– Nouns ratio | 0.222 ± 0.078 | 0.216 ± 0.049 | 0.271 ± 0.059 | 0.01* | 0.066 | — | — | — |
| SAND Picture description –– Verbs ratio | 0.169 ± 0.038 | 0.173 ± 0.036 | 0.175 ± 0.038 | 0.88 | 0.91 | — | — | — |
| SAND Picture description –– S N. sentences | 8.68 ± 5.24 | 7.70 ± 3.87 | 10.11 ± 7.43 | 0.66 | 0.76 | — | — | — |
| SAND Picture description –– Subordinates ratio | 0.426 ± 0.439 | 0.539 ± 1.055 | 0.488 ± 1.145 | 0.14 | 0.26 | — | — | — |
| SAND Picture description –– Repaired sequences ratio | 0.195 ± 0.430 | 0.061 ± 0.055 | 0.078 ± 0.061 | 0.64 | 0.76 | — | — | — |
| SAND Picture description –– Phonological errors ratio | 0.002 ± 0.006 | 0.005 ± 0.011 | 0.011 ± 0.020 | 0.13 | 0.26 | — | — | — |
| SAND Picture description –– Lexical/formal errors ratio | 0.087 ± 0.154 | 0.036 ± 0.032 | 0.027 ± 0.031 | 0.094 | 0.22 | — | — | — |

svPPA: semantic primary progressive aphasia; lvPPA: logopenic primary progressive aphasia; nfvPPA: non-fluent/agrammatic primary progressive aphasia; MMSE: Mini-Mental State Examination; FAB: Frontal Assessment Battery; TMT: Trail-Making Test; RAVLT: Rey Auditory Verbal Learning Test; SAND: Screening for Aphasia in NeuroDegeneration.

**Table S4 Statistical results of acoustic and linguistic features**

| **Features** | **svPPA** | **lvPPA** | **nfvPPA** | **p-value** | **p-values corrected** | **p-value svPPA vs. lvPPA** | **p-value svPPA vs. nfvPPA** | **p-value lvPPA vs. nfvPPA** |
| --- | --- | --- | --- | --- | --- | --- | --- | --- |
| Adjective rate | 0.023 ± 0.017 | 0.024 ± 0.021 | 0.016 ± 0.017 | 0.24 | 0.39 | - | **-** | **-** |
| Adjectives | 3.14 ± 3.05 | 2.68 ± 3.59 | 1.22 ± 1.59 | 0.012* | 0.040* | - | 0.015* | 0.039* |
| Adpositions | 12.2 ± 6.61 | 8.76 ± 5.79 | 6.70 ± 5.65 | 0.0041** | 0.016* | 0.08 | 0.0027** | 0.091 |
| Adverb rate | 0.11 ± 0.04 | 0.096 ± 0.050 | 0.053 ± 0.044 | <0.001*** | 0.0029** | 0.44 | <0.001*** | 0.0027** |
| Adverbs | 16.77 ± 15.02 | 10.08 ± 8.86 | 4.52 ± 5.46 | <0.001*** | 0.0029** | 0.084 | <0.001*** | 0.0048** |
| Articulation Duration (VAD) | 65.29 ± 39.87 | 57.31 ± 31.84 | 50.76 ± 21.84 | 0.542 | 0.70 | - | - | - |
| Articulation Rate (ASR) | 151 ± 45 | 119 ± 44 | 99 ± 37 | <0.001*** | 0.0029** | 0.009** | <0.001*** | 0.193 |
| Articulation Rate (VAD) | 147.7 ± 88.00 | 131.55 ± 141.10 | 91.23 ± 25.32 | <0.001*** | 0.0033** | 0.041* | <0.001*** | 0.037* |
| Closed class rate | 0.519 ± 0.041 | 0.516 ± 0.045 | 0.517 ± 0.066 | 0.972 | 0.99 | - | - | - |
| Closed Class Words | 68.55 ± 35.46 | 50.11 ± 26.98 | 38.87 ± 22.24 | <0.001*** | 0.0053** | 0.051 | <0.001*** | 0.051 |
| Content word percentage | 47.88 ± 4.99 | 47.71 ± 7.18 | 48.05 ± 9.46 | 0.972 | 0.99 | - | - | - |
| Content Words | 64.91 ± 38.61 | 46.35 ± 23.62 | 35.09 ± 18.06 | 0.002** | 0.0091** | 0.083 | 0.0013** | 0.058 |
| Determiner rate | 0.158 ± 0.062 | 0.186 ± 0.057 | 0.212 ± 0.098 | <0.001*** | 0.0033** | 0.177 | <0.001*** | 0.017* |
| Determiners | 20.32 ± 9.68 | 16.57 ± 6.35 | 15.30 ± 6.02 | 0.067 | 0.15 | - | - | - |
| F0 Mean | 155.21 ± 30.99 | 152.36 ± 37.87 | 151.10 ± 37.14 | 0.928 | 0.99 | - | - | - |
| F0 Median | 143.63 ± 31.57 | 145.17 ± 38.07 | 146.83 ± 39.45 | 0.982 | 0.99 | - | - | - |
| F0 Range | 492.19 ± 141.39 | 524.89 ± 146.91 | 450.42 ± 147.65 | 0.179 | 0.31 | - | - | - |
| F0 Std | 50.90 ± 27.22 | 42.85 ± 13.89 | 39.16 ± 14.57 | 0.531 | 0.70 | - | - | - |
| HNR Mean | 6.48 ± 2.21 | 7.79 ± 2.46 | 7.35 ± 2.42 | 0.143 | 0.29 | - | - | - |
| HNR Std | 5.03 ± 0.64 | 4.94 ± 0.77 | 4.95 ± 0.67 | 0.812 | 0.89 | - | - | - |
| Intransitive verb rate | 0.075 ± 0.033 | 0.083 ± 0.036 | 0.056 ± 0.033 | 0.021* | 0.062 | - | - | - |
| Intransitive Verbs | 10.73 ± 7.24 | 7.84 ± 4.68 | 4.17 ± 2.81 | <0.001*** | 0.0032** | 0.148 | <0.001*** | 0.0070** |
| Jitter Local | 0.0306 ± 0.0059 | 0.0274 ± 0.0039 | 0.0258 ± 0.0077 | 0.0075** | 0.027* | 0.12 | 0.0054** | 0.12 |
| Jitter Local Absolute | 0.000201 ± 0.000046 | 0.000189 ± 0.000053 | 0.000175 ± 0.000055 | 0.174 | 0.31 | - | - | - |
| Mean Articulation Duration (VAD) | 5.33 ± 4.29 | 4.43 ± 3.43 | 3.30 ± 1.89 | 0.305 | 0.26 | - | - | - |
| Mean IWI | 0.394 ± 0.199 | 0.479 ± 0.255 | 0.651 ± 0.322 | 0.0064** | 0.024* | 0.222 | 0.0056** | 0.048* |
| Mean Pause Duration (ASR) | 0.634 ± 0.367 | 0.818 ± 0.538 | 1.068 ± 0.755 | 0.049* | 0.12 | - | - | - |
| Mean Pause Duration (VAD) | 3.04 ± 3.29 | 2.48 ± 2.29 | 2.43 ± 1.44 | 0.935 | 0.99 | - | - | - |
| Mean Word Duration | 0.420 ± 0.110 | 0.612 ± 0.350 | 0.727 ± 0.355 | <0.001*** | 0.0043** | 0.019* | <0.001*** | 0.099 |
| Mean words per sentence | 19.55 ± 9.90 | 17.72 ± 10.31 | 19.63 ± 19.36 | 0.373 | 0.57 | - | - | - |
| Normalized IWI | 0.925 ± 0.371 | 0.802 ± 0.285 | 0.972 ± 0.462 | 0.324 | 0.51 | - | - | - |
| Noun Count | 27.36 ± 13.20 | 21.22 ± 8.86 | 21.04 ± 9.10 | 0.172 | 0.31 | - | - | - |
| Noun rate | 0.220 ± 0.058 | 0.232 ± 0.054 | 0.298 ± 0.055 | <0.001*** | 0.0027** | 0.30 | <0.001*** | <0.001*** |
| Noun to pronoun ratio | 1.73 ± 0.71 | 2.28 ± 1.34 | 4.62 ± 4.67 | 0.029* | 0.078 | - | - | - |
| Noun to verb ratio | 1.69 ± 0.91 | 1.97 ± 0.94 | 3.23 ± 2.33 | 0.0011** | 0.0058** | 0.254 | 0.0012** | 0.0098** |
| Num Long Pauses (ASR) | 20 ± 10.72 | 17.43 ± 7.96 | 17.43 ± 6.70 | 0.725 | 0.82 | - | - | - |
| Num Long Pauses (VAD) | 11.50 ± 7.22 | 13.11 ± 7.26 | 13.65 ± 5.86 | 0.531 | 0.70 | - | - | - |
| Num Pauses (ASR) | 83.55 ± 46.54 | 61.05 ± 34.58 | 50.22 ± 28.58 | 0.0085** | 0.029* | 0.09 | 0.0064** | 0.156 |
| Num Pauses (VAD) | 13.64 ± 8.19 | 15.19 ± 8.28 | 16.35 ± 7.25 | 0.457 | 0.66 | - | - | - |
| Num Short Pauses (ASR) | 63.55 ± 38.03 | 43.62 ± 29.50 | 32.78 ± 23.41 | 0.0041** | 0.016* | 0.06 | 0.0029** | 0.133 |
| Num Short Pauses (VAD) | 2.14 ± 2.49 | 2.08 ± 2.15 | 2.70 ± 2.80 | 0.618 | 0.75 | - | - | - |
| Number of sentences | 7.77 ± 4.09 | 6.16 ± 2.96 | 5.96 ± 4.66 | 0.154 | 0.29 | - | - | - |
| Percent Pause Time (ASR) | 43.74 ± 7.88 | 40.37 ± 8.67 | 44.50 ± 10.79 | 0.19 | 0.33 | - | - | - |
| Percent Pause Time (VAD) | 35.03 ± 23.00 | 37.51 ± 22.09 | 41.34 ± 19.96 | 0.52 | 0.70 | - | - | - |
| Preposition rate | 0.095 ± 0.033 | 0.088 ± 0.034 | 0.081 ± 0.037 | 0.413 | 0.62 | - | - | - |
| Pronoun rate | 0.139 ± 0.036 | 0.126 ± 0.047 | 0.110 ± 0.070 | 0.201 | 0.34 | - | - | - |
| Pronouns | 18.09 ± 10.15 | 12.78 ± 8.55 | 8.96 ± 8.51 | 0.0019** | 0.0090** | 0.096 | 0.0012** | 0.096 |
| Shimmer Local | 0.164 ± 0.019 | 0.156 ± 0.021 | 0.148 ± 0.022 | 0.04* | 0.10 | - | - | - |
| Shimmer Local dB | 1.468 ± 0.123 | 1.417 ± 0.146 | 1.371 ± 0.148 | 0.07 | 0.15 | - | - | - |
| Speech Rate (ASR) | 80.71 ± 27.89 | 64.04 ± 25.27 | 49.45 ± 18.44 | <0.001*** | 0.0032** | 0.038* | <0.001*** | 0.075 |
| Std Articulation Duration (VAD) | 4.54 ± 4.33 | 3.69 ± 3.60 | 2.62 ± 2.19 | 0.472 | 0.31 | - | - | - |
| Std IWI | 1.068 ± 0.859 | 1.100 ± 0.705 | 1.349 ± 0.900 | 0.221 | 0.36 | - | - | - |
| Std Pause Duration (ASR) | 1.34 ± 1.17 | 1.36 ± 0.96 | 1.62 ± 1.22 | 0.43 | 0.63 | - | - | - |
| Std Pause Duration (VAD) | 2.22 ± 1.83 | 1.89 ± 1.49 | 2.40 ± 2.00 | 0.789 | 0.87 | - | - | - |
| Std Word Duration | 0.752 ± 0.462 | 1.084 ± 0.730 | 1.362 ± 0.940 | 0.034* | 0.086 | - | - | - |
| Std words per sentence | 11.35 ± 7.64 | 9.67 ± 5.92 | 7.22 ± 5.05 | 0.126 | 0.26 | - | - | - |
| Total IWI | 44.68 ± 18.63 | 41.49 ± 21.31 | 41.74 ± 17.36 | 0.677 | 0.78 | - | - | - |
| Total Pause Time (ASR) | 43.39 ± 18.16 | 40.61 ± 21.08 | 41.20 ± 17.15 | 0.68 | 0.78 | - | - | - |
| Total Pause Time (VAD) | 32.12 ± 24.71 | 34.37 ± 26.09 | 36.26 ± 20.14 | 0.592 | 0.73 | - | - | - |
| Total Speech Duration | 100.50 ± 41.26 | 97.18 ± 37.72 | 91.14 ± 23.70 | 0.676 | 0.78 | - | - | - |
| Total Articulation Duration (ASR) | 53.58 ± 25.40 | 51.73 ± 19.35 | 46.32 ± 13.90 | 0.633 | 0.76 | - | - | - |
| Transitive verb rate | 0.051 ± 0.020 | 0.050 ± 0.024 | 0.060 ± 0.033 | 0.54 | 0.70 | - | - | - |
| Transitive Verbs | 6.91 ± 4.50 | 4.54 ± 2.79 | 4.13 ± 2.35 | 0.023* | 0.065 | - | - | - |
| Transitive-intransitive verb ratio | 0.849 ± 0.735 | 0.801 ± 0.755 | 0.917 ± 0.825 | 0.575 | 0.73 | - | - | - |
| Verb Count | 17.64 ± 10.99 | 12.38 ± 6.22 | 8.30 ± 4.36 | <0.001*** | 0.0053** | 0.074 | <0.001*** | 0.035* |
| Verb rate | 0.127 ± 0.041 | 0.132 ± 0.038 | 0.116 ± 0.040 | 0.488 | 0.69 | - | - | - |
| Word Count | 133.45 ± 73.54 | 96.46 ± 49.92 | 73.96 ± 39.09 | 0.0011** | 0.0058** | 0.073 | <0.001*** | 0.073 |

svPPA: semantic primary progressive aphasia; lvPPA: logopenic primary progressive aphasia; nfvPPA: non-fluent/agrammatic primary progressive aphasia; VAD: voice activity detection; ASR: automatic speech recognition; IWI: inter-word interval; HNR: harmonic-to-noise ratio.

**Extended feature set analysis**

**Methods**

In addition to the acoustic and linguistic features extracted in the main analysis, other syntactic and morphosyntactic complexity measures, and lexical frequency features were considered, specifically, (clauses per sentence, subordinate clause ratio, mean dependency distance, high- and low- frequency verb rates).

**Results**

a) **Binary classification**

For the binary classification, the Fusion modality yielded the highest performance, achieving 78.33% accuracy, 78.62% ROC-AUC, 89.49% specificity, and 65.22% sensitivity. While Acoustic modality achieved the lowest classification accuracy (the same as the main analysis), the Linguistic strategy achieved an accuracy of 68.33%, with a ROC-AUC of 66.27%. Finally, the Multimodal approach showed an accuracy of 73.33%, a ROC-AUC of 75.56%, with the best sensitivity of 73.91%. The most predictive features remain the same as for the main analysis (e.g, noun rate and count, local jitter, and articulation rate), with clause per sentence and subordinate clause ratio being selected in at least one fold.

b) **Multiclass classification**

For the multiclass classification, the Acosuitc modality yielded the highest performance, achieving 61.45% accuracy and 70.37% ROC-AUC. Instead, the overall performance of Fusion modality achieved an accuracy of 55.42%, with a ROC-AUC of 70.71%. The Linguistic and Multimodal approaches achieved lower results, showing an overall accuracy of 40.96% for Linguistic and 51.81% for Multimodal, and ROC-AUC of 60.01% and 70.80%, respectively. For the class-specific metrics, Acoustic modality showed the best performance for both svPPA and lvPPA, yielding precision, specificity, and sensitivity values of 53.85%, 80.00%, ad 60.87% for svPPA, and 67.57%, 73.91%, and 67.57% for lvPPA, respectively. For nfvPPA, the Fusion modality achieved the highest precision and specificity, reaching 63.16% and 88.33% respectively, vehicle sharing the highest sensitivity with the Acoustic modality at 52.71%. The features that mainly contributed to the classification in the best modality were jitter local and articulation rate from VAD. Moreover, pause and IWI metrics contributed to distinguishing the three classes.
